# COVID-19 and NSTEMI Outcomes among Hospitalized Patients in the United States and Racial Disparities in Mortality: Insight from National Inpatient Sample Database

**DOI:** 10.3390/vaccines10122024

**Published:** 2022-11-26

**Authors:** Harris Majeed, Karthik Gangu, Shazib Sagheer, Ishan Garg, Umair Khan, Hina Shuja, Aniesh Bobba, Prabal Chourasia, Rahul Shekhar, Sindhu Reddy Avula, Abu Baker Sheikh

**Affiliations:** 1 Department of Internal Medicine, University of New Mexico Health Sciences Center, Albuquerque, NM 87106, USA; 2 Department of Internal Medicine, University of Kansas Medical Center, Kansas City, KS 66103, USA; 3 Division of Cardiology, University of New Mexico Health Sciences Center, Albuquerque, NM 87106, USA; 4 Department of Medicine, Karachi Medical and Dental College, Karachi 74700, Pakistan; 5 Department of Medicine, John H. Stronger Hospital, Cook County, Chicago, IL 60612, USA; 6 Division of Hospital Medicine, Mary Washington Hospital, Fredericksburg, VA 22401, USA; 7 Department of Interventional Cardiology, Division of Cardiology, University of Kansas, St. Francis Campus, Kansas City, KS 66606, USA

**Keywords:** COVID-19, NSTEMI, United States, mortality, national inpatient sample

## Abstract

The COVID-19 pandemic has impacted healthcare delivery to patients with non-ST-segment elevation myocardial infraction (NSTEMI). The aim of our retrospective study is to determine the effect of COVID-19 on inpatient NSTEMI outcomes and to investigate whether changes in cardiac care contributed to the observed outcomes. After multivariate adjustment, we found that NSTEMI patients with COVID-19 had a higher rate of inpatient mortality (37.3% vs. 7.3%, adjusted odds ratio: 4.96, 95% CI: 4.6–5.4, *p* < 0.001), increased length of stay (9.9 days vs. 5.4 days, adjusted LOS: 3.6 days longer, *p* < 0.001), and a higher cost of hospitalization (150,000 USD vs. 110,000 USD, inflation-adjusted cost of hospitalization: 36,000 USD higher, *p* < 0.001) in comparison to NSTEMI patients without COVID-19, despite a lower burden of pre-existing cardiac comorbidity. NSTEMI patients with COVID-19 also received less invasive cardiac procedures (coronary angiography: 8.7% vs. 50.3%, *p* < 0.001; PCI: 4.8% vs. 29%, *p* < 0.001; and CABG: 0.7% vs. 6.2%, *p* < 0.001). In our study, we observed increased mortality and in-hospital complications to be a combined effect of COVID-19 infection and myocardial inflammation as a result of cytokine storm, prothrombic state, oxygen supply/demand imbalance and alterations in healthcare delivery from January to December 2020.

## 1. Introduction

Acute non-ST-segment elevation myocardial infarction (NSTEMI) is a cardiac condition that requires timely evaluation and management [1]. The coronavirus disease-2019 (COVID-19) pandemic, caused by severe acute respiratory syndrome-coronavirus-2 (SARS-CoV2), has adversely impacted the delivery of healthcare to patients who present with NSTEMI [2]. Extensive care process changes were observed across the world during this period, including alternative cardiac reperfusion protocols, supply chain shortages, redirection of resources, and delays in patient presentation and intervention [3]. This observation is complicated by the fact that COVID-19 patients have more cardiovascular sequalae from primary SARS-COV2 infection, including coronary thrombosis, cardiomyopathy, myocarditis, and pericarditis [4,5]. There are limited data regarding NSTEMI mortality in hospitalized COVID-19 patients during the early pandemic. The aim of our study is twofold: (1) to determine the consequence of COVID-19 infection on inpatient NSTEMI mortality, and (2) to investigate whether changes to the delivery of cardiac care contributed to the observed outcomes using a large, representative sample from the United States based National Inpatient Sample (NIS) database.

## 2. Materials and Methods

This retrospective study utilized the NIS Healthcare Cost Utilization Project (HCUP) sponsored by the Agency for Healthcare and Research and Quality (AHRQ) database, which is an all-payer database that approximates a 20% stratified sample of discharges from US community hospitals [6]. In this analysis, we used the 2020 NIS dataset, which included hospitalization from January 1, 2020, to December 31, 2020, and was made available to the public in October 2022. All patients who were 18 years of age and older and were admitted to the hospital with NSTEMI and COVID-19 infection were included in this study. International classification of diseases 10th—clinical modification (ICD-10-CM) codes were used to retrieve patient samples with comorbid conditions and ICD-10 procedure codes were used to identify inpatient procedures. A detailed code summary is provided in Supplementary Table S1. Patients who were under the age of 18 years or were transferred out of the index hospital were excluded from this study.

### 2.1. Covariates

The NIS database contains data regarding in-hospital outcomes, procedures, and other discharge-related information. Patient-related variables, hospital-related variables, and indicators of illness severity were included, as below:

- Patient: age, race, sex, comorbidities, insurance status, mean income in patient’s zip code, and disposition.
- Hospital: teaching status, bed size, and region.
- Illness severity: length of stay (LOS), mortality, cost of hospitalization, Elixhauser comorbidity index [7], in-hospital complications, mechanical ventilation, circulatory support, and vasopressor use.

### 2.2. Study Outcomes

The primary outcome was in-hospital mortality.

Secondary outcomes included:

- Intubation and vasopressor use.
- Utilization of percutaneous left ventricular assist device (pLVAD) and intra-aortic balloon pump (IABP).
- Rates of coronary revascularization with percutaneous coronary intervention (PCI), coronary artery bypass grafting (CABG), and systemic thrombolytic therapy.
- Length of stay (LOS).
- Cost of hospitalization.

### 2.3. In-Hospital Complications

Complications associated with NSTEMI were sorted into mechanical, arrhythmic, and inflammatory. ICD-10 codes were used to identify these comorbidities.

- Mechanical: cardiogenic shock.
- Arrhythmic: atrial fibrillation, atrial flutter, ventricular tachycardia, ventricular fibrillation, sinus bradycardia, first-degree atrioventricular nodal block, second-degree atrioventricular nodal block, third-degree atrioventricular nodal block, transvenous pacing, and permanent pacemaker implantation.
- Inflammatory: pericarditis, myocarditis.

### 2.4. Statistical Methods

STATA 17 (StataCorp LLC, College Station, TX) was utilized for statistical analysis. The unweighted sample was 6.47 million observations, and the weighted sample was 32.3 million discharges for the year 2020. Patients who were admitted with NSTEMI were retrieved with ICD-10-CM codes, and this group was further divided based on COVID-19 status. Chi-square test was used to compare categorical variables and linear regression was used for continuous variables. For the primary outcome, univariate logistic regression was used to calculate the unadjusted odds ratio for variables of interest and a *p* value of <0.2 was used for univariate logistic regression to build a multivariate logistic regression model to adjust for potential confounders [8]. We arbitrarily chose *p* < 0.2 because covariates with *p*-values closer to 0.05 have a higher chance of elucidating the strength of the exposure variable and will narrow the standard error of exposure. By setting *p*-values on the univariate screen to significance (<0.05), there is a possibility of missing potential confounders or covariates that can impact the standard error for exposure variable. The multivariate linear regression model used for continuous variables (LOS and cost of hospitalization) utilized a two-tailed *p*-value of 0.05 to determine significance.

## 3. Results

### 3.1. Baseline Characteristics

We identified 515,565 patients with NSTEMI between January 1, 2020 and December 31, 2020. Of these, 23,521 patients (4.6%) had a diagnosis of COVID-19. NSTEMI patients with COVID-19 were significantly older (61.4% were above the age of 70 years vs. 51.2%, *p* < 0.001), had a greater proportion of Hispanics (20.5% vs. 9.5%, *p* < 0.001) or African Americans (18.5% vs. 13.4%, *p* < 0.001), and were more likely to have household incomes below 50,000 USD (37.4% vs. 31.7%, *p* < 0.001) when compared to NSTEMI patients without COVID-19.

NSTEMI patients with COVID-19 had a lower prevalence of cardiac comorbidities when compared to NSTEMI patients without COVID-19, including coronary artery disease (45.4% vs. 67.4%, *p* < 0.001), congestive heart failure (47.3% vs. 50.8%, *p* < 0.001), uncomplicated hypertension (27.1% vs. 32.1%, *p* < 0.001), smoking (25.4% vs. 43.8%, *p* < 0.001), and obesity (19.7% vs. 21.5%, *p* = 0.005). Additionally, NSTEMI patients with COVID-19 also had more chronic pulmonary disease (24% vs. 25.5%, *p* = 0.02). Conversely, NSTEMI patients with COVID-19 had a higher prevalence of complicated diabetes mellitus (39% vs. 32.4%, *p* < 0.001) and renal failure (38.3% vs. 32.5%, *p* < 0.001). There were no significant differences in the rates of complicated hypertension or uncomplicated diabetes mellitus in the two groups. While there was variability in the geographic distribution of the patients, there were no significant differences in the hospital size and type, in that most NSTEMI patients, regardless of COVID-19 status, were seen at urban teaching hospitals (68.9% vs. 69.7%, *p* = 0.46). NSTEMI patients with COVID-19 had a higher proportion of Medicare beneficiaries (70.9% vs. 65.3%, *p* < 0.001) and a lower proportion of private health insurance beneficiaries (16.7% vs. 20.7%, *p* < 0.001) when compared to the non-COVID-19 cohort. Table 1 outlines the baseline characteristics of both study cohorts.

### 3.2. In-Hospital Mortality and Secondary Non-Procedural Outcomes

After multivariate adjustment, we found that the in-hospital mortality of NSTEMI patients was significantly higher among COVID-19 patients (37.3% vs. 7.3%, adjusted odds ratio: 5, 95% CI: 4.6–5.4, *p* < 0.001); this group also had an increased length of stay (9.9 days vs. 5.4 days, adjusted LOS: 3.6 days longer, *p* < 0.001) and a higher cost of hospitalization (149,121 USD vs. 110,503 USD, inflation-adjusted cost of hospitalization: 36,136 USD higher, *p* < 0.001). Of those patients who survived, fewer NSTEMI patients with COVID-19 were able to return home (41.6% vs. 61.1%, *p* < 0.001) and more required skilled nursing or long-term acute care (38.8% vs. 17.4%, *p* < 0.001) when compared to NSTEMI patients without COVID-19. Table 2 outlines the primary and secondary non-procedural outcomes of both study cohorts.

For reference, the mortality rate of COVID-19 patients without NSTEMI during this same period was 12.5%. Moreover, to account for the month-to-month variability in COVID-19 disease burden and the logistical challenges during the early pandemic, we examined monthly mortality among NSTEMI patients with COVID-19, as outlined in Table 3. After multivariate regression, we found no difference in mortality during each month of the pandemic in 2020. Notably, while the earliest reported COVID-19 cases in the United States were in late January 2020, we restricted our analysis to March 2020 onwards, since our study relies on ICD-10 codes, which were not reliably available during the early pandemic.

We also performed a subgroup analysis of mortality and found that, among NSTEMI with COVID-19, Hispanics (20.5% vs. 9.4%, *p* < 0.001), African Americans (18.1% vs. 12.7%, *p* < 0.001), and Asian or Pacific Islanders (4.4% vs. 3.7%, *p* < 0.001) had an increased in-hospital mortality when compared to the NSTEMI without COVID-19 cohort. Conversely, a significantly lower percentage of Caucasians in the NSTEMI with COVID-19 cohort had inpatient mortality (47.6% vs. 70.7%, *p* < 0.001). Table 4 outlines mortality subgroup analysis.

### 3.3. Invasive Cardiac Procedures and Cardiac Complications

NSTEMI patients with COVID-19 required higher rates of mechanical ventilation (37.1% vs. 13.4%, *p* < 0.001) and vasopressors (6.5% vs. 2.5%, *p* < 0.001), despite there being no difference in rates of cardiogenic shock (4.7% vs. 4.5%, *p* = 0.56). These patients also had significantly higher rates of atrial fibrillation (3.7% vs. 3.2%, *p* < 0.001) and ventricular tachycardia (7% vs. 5.8%, *p* < 0.001). Most notably, NSTEMI patients with COVID-19 were significantly less likely to receive all types of invasive cardiac interventions including coronary angiography (8.7% vs. 50.3%, *p* < 0.001), percutaneous coronary interventions (4.8% vs. 29%, *p* < 0.001), coronary artery bypass grafting (0.7% vs. 6.2%, *p* < 0.001), intra-aortic balloon pumps (0.6% vs. 1.7%, *p* < 0.001), percutaneous left ventricular assist devices (0.2% vs. 0.8%, *p* < 0.001), transvenous pacing (0.2% vs. 1.0%, *p* < 0.001), and permanent pacemaker implantation (0.3% vs. 0.7%, *p* < 0.001) when compared to NSTEMI patients without COVID-19. There was no difference in the use of systemic thrombolytic therapy (0.6% vs. 0.7%, *p* = 0.59) in NSTEMI patients among both groups. Table 5 outlines the distribution of procedures and cardiac complications of both study cohorts.

## 4. Discussion

In our study, NSTEMI patients with concomitant COVID-19 infection had a higher rate of inpatient mortality than those without COVID-19 infection. They had an accompanying increase in length of stay and cost of hospitalization. COVID-19 patients also received significantly fewer invasive cardiac (diagnostic, definitive, and temporizing) procedures. To our knowledge, this is the largest study examining NSTEMI mortality in patients with documented COVID-19 infection. Our results are a significant departure from the existing literature; a meta-analysis of studies examining inpatient NSTEMI mortality during 2020 found a non-significant increase in mortality without stratification by COVID-19 status (OR: 1.39, 95% CI: 0.95–2.04) [9]. To rule out the possibility that month-dependent factors such as staffing and changes in COVID treatment confounded these observed differences in mortality, we conducted a multivariate regression for monthly mortality breakdown. We found no difference in inpatient mortality each month of the pandemic in 2020.

In the early pandemic, the existing literature confirms the decline in invasive catheterization among NSTEMI patients. A large, retrospective British study found a 45% decrease in PCI procedures from the first 10 weeks of 2020 to the remainder of the year after the lockdown was instituted for the COVID-19 pandemic [10]. The limitation of existing studies is the utilization of time periods rather than patient-specific COVID-19 diagnosis for analysis, which is a distinct strength of our study. There are clinical markers in our dataset indicating that the NSTEMI with COVID-19 cohort was more critically ill, with higher levels of vasopressor and mechanical ventilatory dependence than the NSTEMI without COVID-19 cohort. Moreover, higher mortality was observed among the NSTEMI patients with COVID-19, despite the lower underlying cardiac and pulmonary comorbidities.

There is a growing body of literature establishing the role of acute SARS-COV2 infection in the development of acute myocardial complications, which include various forms of myocardial injury (myocarditis, stress cardiomyopathy, and myocardial infarction), congestive heart failure, and cardiac arrhythmias [11,12]. The proposed mechanisms of COVID-19 mediated cardiovascular pathophysiology include [13,14,15]:

- Myocardial injury from hemodynamic instability or hypoxemia.
- Inflammatory myocarditis.
- Stress cardiomyopathy.
- Microvascular dysfunction.
- Thrombosis with coronary artery plaque destabilization due to inflammatory hypercoagulability.

COVID-19 patients without NSTEMI had an in-hospital mortality rate of 12.5%, which is higher than NSTEMI patients without COVID-19 (in-hospital mortality of 7.3%), but significantly lower than NSTEMI patients with COVID-19 (in-hospital mortality of 37.3%). In summary, while COVID-19 results in overall increased morality, there is an additive effect in NSTEMI patients, whereby the mortality is significantly higher among NSTEMI patients with COVID-19 patients. We believe that the etiology of the increased mortality in NSTEMI patients with COVID-19 is multifactorial; these patients were more critically ill, likely due to acute COVID-19 infection, and were at higher risk of cardiovascular complications. Additionally, myocardial inflammation, the direct cytotoxic effect of virus on myocytes, cytokinin storm, oxygen supply demand mismatch and prothrombotic state, might have a compounded effect on the excess mortality observered in this cohort. Despite this, it is difficult to ignore the alterations in healthcare delivery during the early COVID-19 pandemic from January to December 2020 as a potential contributor.

Observational studies suggest that COVID-19 increases the risk of acute myocardial infection (AMI); one analysis found that the risk of AMI in patients with COVID-19 is significantly elevated (0.03 versus 0.01 percent; adjusted odds ratio 1.22, 95% CI 1.08–1.38) [16]. There is also evidence of subacute cardiac complications after the first 30 days of SARS-COV2 infection, including increased incidence of dysrhythmias, ischemic and non-ischemic cardiomyopathies with congestive heart failure, peri-myocarditis, and thromboembolic disease [17]. It is also important to note that the observed cardiac complications, and overall mortality during 2020 in the early phase of the pandemic may have been higher due to the lack of COVID-19 therapeutics and vaccinations. At this time, glucocorticoids were underutilized and remdesivir, tocilizumab, and mRNA vaccinations were not widely available; there is evidence to suggest that, during the later course of the pandemic, these agents led to a significantly reduced mortality and morbidity [18].

There is a bidirectional relationship between COVID-19 infection and coronary artery disease (CAD); the risk of severe COVID infection considerably increased in those with pre-existing coronary artery disease [19]. According to a report released by the Center for Disease Control and Prevention (CDC), based on a Chinese patient population in early 2020, 4.2% of all COVID-19 patients had a known diagnosis of coronary heart disease, which increased to 22.7% among fatal cases of COVID-19 [20]. With this mind, it is probable that a portion of COVID-19 patients had undiagnosed CAD disease with an index NSTEMI presentation precipitated by SARS-COV2 infection, which may account for a portion of the mortality difference.

With respect to the lower utilization of cardiac procedures and its relation to the observed increase in mortality, there are several possible explanations. Firstly, we believe that a major contributing factor is the strain of the COVID-19 pandemic on systems of healthcare delivery [21]. Throughout various stages of the pandemic, there have been critical supply shortages; regarding NSTEMI care, the shortages in heparin, iodinated contrast, anesthetic agents for moderate sedation (midazolam and fentanyl citrate specifically), troponin assays, SARS-COV2 rapid PCR testing, N95 respirators, and personal protective equipment were thought to be particularly impactful for cardiac catheterization and other bedside cardiac procedures [22,23,24]. Similarly, the redirection of inpatient resources, catheterization lab unavailability, and ancillary staffing deficiencies are notable contributors [25]. A secondary explanation for the decreased cardiac procedural utilization is that the referring clinicians and cardiologists may have felt that COVID-19 patients were less likely to benefit from invasive cardiac interventions due to poorer prognosis from acute infection. Correspondingly, patients with critical illness due to COVID-19, commonly characterized by hemodynamic instability, circulatory shock, and respiratory failure with mechanical ventilation, are suboptimal surgical candidates for CABG and, to a lesser extent, for PCI. While diagnostic errors are possible, the similar rates of systemic thrombolytic therapy among the COVID-19 and non-COVID-19 groups suggest that obstructive NSTEMI remained on the differential for these patients.

We also found both economic and racial disparities in mortality. Hispanics, African Americans, and those with lower household income had increased mortality with NSTEMI and COVID-19 infection, which is consistent with the prior literature [26]. As expected, NSTEMI mortality increased with age, regardless of COVID-19 status. Mortality was also significantly higher at large, urban teaching hospitals, irrespective of COVID-19 status, and is likely an indicator of the acuity and comorbidity burden of this patient population [27]. We observed a higher inpatient NSTEMI mortality in Caucasians without COVID-19 in comparison to those with COVID-19; this novel finding is distinct from the previous literature and presents an opportunity for further analysis [28,29]. At present, there is no evidence to suggest that COVID-19 infection is protective or prevents mortality in NSTEMI patients who are Caucasian, and our finding is likely more reflective of the poorer outcomes in Caucasian NSTEMI patients overall.

Our study has several limitations. The first is potential detection bias, particularly as this relates to COVID-19 infection. The ICD-10 diagnosis code for COVID-19 was not developed until late 2020, which allows for the possibility that certain COVID-19 diagnoses were not captured in our dataset. Likewise, COVID-19 testing remained in short supply and the NIS database does not provide laboratory test results to confirm COVID-19 status. This principle applies, to a lesser extent, to incompletely or inaccurately documented comorbid diagnoses. The second limitation is that selection bias and confounding remain a possibility in this retrospective study. Additionally, it is not possible to differentiate the proportion of type I from type 2 NSTEMI in this study. Our statistical analysis accounted for numerous demographic and clinical factors, including existing medical comorbidity, via multivariable logistic regression—however, unaccounted variables may underlie our observed outcomes. This dataset did not include biochemical profiles, physical examination, or radiographic markers of COVID-19 severity, which may conceivably have contributed more heavily to the observed mortality than an NSTEMI diagnosis.

## 5. Conclusions

We conclude that NSTEMI patients with concomitant COVID-19 infection were nearly five times as likely to die in the hospital in comparison with NSTEMI patients without COVID-19 infection. This finding is driven by both clinical complications of acute COVID-19 infection and system-wide alterations in cardiac care delivery due to the healthcare burden of the COVID-19 pandemic. Based on our study, we recommend further examination of the cardiac care delivery process during the early pandemic. Future investigations should evaluate process measures that explore time-to-intervention, procedural complications, and barriers to definitive therapies, such as PCI and CABG. Lastly, in addition to focusing on risk-mitigation strategies for infection prevention during future pandemics, it is equally important to develop strategies to maintain critical processes to allow for the delivery of healthcare to patients with life-threatening conditions such as NSTEMI, in order to reduce unnecessary morbidity and mortality.

## Figures and Tables

**Table 1:** Baseline NSTEMI Patient Characteristics Based on COVID-19 Status.

Characteristics	+COVID 19	−COVID 19	*p*-Value
Total = 515,565	n = 23,521 (4.56%)	n = 492,044 (95.44%)	
Female Sex	39.92%	40.82%	0.21
Age Groups			<0.001
≥18–29	0.6%	0.44%	
30–49	5.46%	8%	
50–69	32.53%	40.35%	
≥70	61.42%	51.21%	
Race			<0.001
Caucasian	51.35%	70.72%	
African American	18.52%	13.35%	
Hispanic	20.52%	9.5%	
Asian Or Pacific Islander	4.36%	3.04%	
Native American	0.72%	0.49%	
Other	4.53%	2.9%	
Median Household Income			<0.001
<49,999 USD	37.42%	31.66%	
50,000–64,999 USD	26.52%	27.71%	
65,000–85,999 USD	21.57%	22.64%	
>86,000 USD	14.49%	17.99%	
Insurance Status			
Medicare	70.87%	65.33%	
Medicaid	9.75%	9.98%	
Private	16.73%	20.71%	
Self-Pay	2.66%	3.97%	
Hospital Size			0.02
Small	24.34%	22.37%	
Medium	31.8%	31.21%	
Large	43.86%	46.41%	
Hospital Teaching Status			0.46
Rural	9.84%	9.15%	
Urban Non-Teaching	21.26%	21.19%	
Urban Teaching	68.9%	69.66%	
Medical Comorbidities			
Coronary Artery Disease	45.39%	67.43%	<0.001
Congestive Heart Failure	47.32%	50.84%	<0.001
Uncomplicated Hypertension	27.15%	32.16%	<0.001
Complicated Hypertension	54.59%	53.2%	0.06
Uncomplicated Diabetes Mellitus	14.31%	13.86%	0.38
Complicated Diabetes Mellitus	38.99%	32.4%	<0.001
Renal Failure	38.29%	32.5%	<0.001
Chronic Pulmonary Disease	24%	25.47%	0.02
Obesity	19.73%	21.49%	0.005
Smoking	25.47%	43.76%	<0.001

**Table 2:** NSTEMI Primary and Secondary Non-Procedural Outcomes Based on COVID-19 Status.

Outcome	+COVID 19	−COVID 19	*p*-Value
In-Hospital Mortality (n = 44,791)	37.3%	7.32%	
	Adjusted Odds Ratio: 4.96 (95% CI: 4.56–5.4)		<0.001
Mean Cost of Hospitalization (USD)	149,121	110,503	
	Adjusted Total Cost: 36,136 USD Higher		<0.001
Mean Length of Stay (Days)	9.89	5.35	
	Adjusted Length of Stay: 3.57 Days Longer		<0.001
Disposition			<0.001
Home	41.57%	61.07%	
Skilled Nursing Facility or Long-Term Acute Care or Nursing Home	38.77%	17.33%	
Home with Healthcare	17.34%	19.28%	
Against Medical Advice	2.32%	2.32%	

+ = Positive, − = Negative.

**Table 3:** Monthly In-Hospital Mortality Among NSTEMI Patients with COVID-19 During 2020.

Month	aOR (95% CI LL-UL)	*p* Value
March	3.92 (2.56–5.99)	<0.001
April	5.35 (4.11–6.97)	<0.001
May	4.85 (3.5–6.64)	<0.001
June	5.36 (3.69–7.8)	<0.001
July	4.57 (3.47–6.02)	<0.001
August	4.23 (3.15–5.67)	<0.001
September	4.81 (3.45–6.7)	<0.001
October	3.42 (2.56–4.57)	<0.001
November	4.45 (3.54–5.59)	<0.001
December	5.08 (4.2–6.14)	<0.001

**Table 4:** NSTEMI Mortality Sub-Group Analysis Based on COVID-19 Status.

Variable	+COVID 19	−COVID 19	*p*-Value
Total Died (n = 44,791)	n = 8773	n = 36,018	
Sex			<0.001
Male	62.01%	56.36%	
Female	37.99%	43.64%	
Age Groups			
≥18–29	0.23%	0.5%	0.12
30–49	2.68%	4.15%	0.004
50–69	27.84%	27.93%	0.94
≥70	69.25%	67.43%	0.13
Race			
Caucasian	47.65%	70.73%	<0.001
African American	18.1%	12.71%	<0.001
Hispanic	20.53%	9.37%	<0.001
Asian Or Pacific Islander	4.36%	3.73%	<0.001
Native American	0.72%	0.55%	0.20
Other	4.54%	2.93%	<0.001
Hospital Teaching Status			
Rural	8.5%	8.77%	0.71
Urban Non-Teaching	21.85%	20.68%	0.37
Urban Teaching	69.65%	70.55%	0.53

+ = Positive, − = Negative.

**Table 5:** Procedures and Cardiac Complications Among NSTEMI Patients Based On COVID-19 Status.

**Procedure**	**+COVID 19**	**−COVID 19**	***p*** **-Value**
Mechanical Ventilation	37.07%	13.38%	<0.001
Vasopressor	6.51%	2.52%	<0.001
Systemic Thrombolytic Therapy	0.62%	0.68%	0.59
Coronary Angiogram	8.74%	50.27%	<0.001
Coronary Artery Bypass Grafting	0.68%	6.24%	<0.001
Percutaneous Coronary Intervention	4.78%	28.95%	<0.001
Intra-Aortic Balloon Pump	0.55%	1.73%	<0.001
Percutaneous Left Ventricular Assist Device	0.21%	0.77%	<0.001
Transvenous Pacing	0.19%	1.01%	<0.001
Permanent Pacemaker Implantation	0.28%	0.74%	<0.001
Cardiac Complication			
Cardiogenic Shock	4.66%	4.48%	0.56
Atrial Fibrillation	23.3%	18.84%	<0.001
Atrial Flutter	3.72%	3.22%	0.06
Ventricular Fibrillation	1.51%	1.47%	0.80
Ventricular Tachycardia	6.99%	5.77%	<0.001
Sinus Bradycardia	3.55%	3.97%	0.16
First Degree Atrioventricular Block	1.19%	1.8%	0.003
Second Degree Atrioventricular Block	0.45%	0.59%	0.19
Third Degree Atrioventricular Block	0.77%	0.99%	0.12

+ = Positive, − = Negative.

## Data Availability

Not applicable.

## Supplementary Materials

The following are available online at https://www.mdpi.com/article/10.3390/vaccines10122024/s1, Table S1: ICD-10 codes.
