# Supplementary material for: COVID-19 and NSTEMI Outcomes among Hospitalized Patients in the United States and Racial Disparities in Mortality: Insight from National Inpatient Sample Database
Source: Vaccines (Basel). 2022 Nov 26;10(12):2024. doi: 10.3390/vaccines10122024 (PMC9780864; doi:10.3390/vaccines10122024)
Supplement: Supplementary file 1 [file vaccines-10-02024-s001.zip › vaccines-2036452-supplementary.pdf]

**Supplementary Table S1:** ICD-10 codes.	
**Variable**	**ICD-10 CM code**
NSTEMI	I21.4XX
Covid	U071, U00, U49, U50, U85, J1282
Cardiogenic shock	R570
Pericarditis	I24.1
A.fib	I48.0, I48.1, I48.91
A.flutter	I48.3, I48.4, I48.92
V.fib	I49.01, I49.02
Vt	I47.2
Sinus brady	R00.1
First degree av block	I44.0
Second degree av block	I44.1
Third degree av block	I44.2
Smoking	F17.XX, Z87.891
CAD	I25.10, I25.11, I25.118, I25.119, I252, I253, I25.4XX, I25.5, I25.6, I25.8XX, I25.7XX, I25.9XX
CHF, HTN, DM, Renal failure, Chronic pulmonary disease, Obesity	Elixhauser comorbidities were used
**Variable**	**ICD-10 procedure code**
Intubation	5A1945Z, 5A1955Z, 5A1935Z, 5A09357, 5A09457, 5A09557
tPA use	3E03317, 3E04317, 3E05317, 3E06317, 3E08317
Vasopressor use	3E030XZ, 3E033XZ, 3E040XZ, 3E043XZ, 3E050XZ, 3E053XZ, 3E060XZ, 3E063XZ
CABG	0210XX, 0211XX, 0212XX, 0213XX
PCI	0203XX, 02704XX, 02713XX, 02714XX, 02723XX, 02724XX, 02733XX, 02734XX, 02C03XX, 02C04XX, 02C13XX, 02C14XX, 02C23XX, 02C24XX, 02C33XX, 02C34XX
Coronary angiography	B217XX, B218XX, B21FXX, B210XX, B211XX, B212XX, B213XX
IABP	5A02210, 5A02110
IMPELLA	5A02116, 5A0211D, 5A02216, 5A0221D
Transvenous pacing	5A1223Z, 5A1213Z
Pacemaker insertion	0J5604Z, 0JH605Z, 0JH606Z, 0JH60PZ, 0JH63PZ, 0JH80PZ, 0JH83PZ, 0JH634Z, 0JH804Z, 0JH834Z, 0JH635Z, 0JH805Z, 0JH835Z, 0JH636Z, 0JH806Z, 0JH836Z, 02H40NZ, 02H43NZ, 02H44NZ, 02H60NZ, 02H63NZ, 02H64NZ, 02H70NZ, 02H73NZ, 02H74NZ, 02HK0NZ, 02HK3NZ, 02HK4NZ, 02HL0NZ, 02HL3NZ, 02HL4NZ
